# Postnatal depressive symptoms amongst women in Central Vietnam: a cross-sectional study investigating prevalence and associations with social, cultural and infant factors

**DOI:** 10.1186/s12884-015-0662-5

**Published:** 2015-09-30

**Authors:** Linda Murray, Michael P. Dunne, Thang Van Vo, Phuong Nguyen Thi Anh, Nigar G. Khawaja, Thanh Ngoc Cao

**Affiliations:** School of Public Health and Social Work, Queensland University of Technology (QUT), Victoria Park Rd, Kelvin Grove, Queensland 4059 Australia; School of Medicine, University of Tasmania, 1/17 Liverpool St, Hobart, Tasmania 7000 Australia; Institute of Community Health Research, Hue University of Medicine and Pharmacy, 06 Ngo Quyen St, Hue City, Vietnam; School of Psychology and Counseling, Queensland University of Technology (QUT), Victoria Park Rd, Kelvin Grove, Queensland 4059 Australia; Faculty of Public Health Hue University of Medicine and Pharmacy, 06 Ngo Quyen St, Hue City, Vietnam; Hue University of Medicine and Pharmacy, 06 Ngo Quyen St, Hue City, Vietnam

**Keywords:** Postnatal, Depression, South-East-Asia, Vietnam, Cross-sectional

## Abstract

**Background:**

This study investigated the prevalence and socio-cultural correlates of postnatal mood disturbance amongst women 18–45 years old in Central Vietnam. Son preference and traditional confinement practices were explored as well as factors such as poverty, parity, family and intimate partner relationships and infant health.

**Methods:**

A cross-sectional study was conducted in twelve randomly selected Commune Health Centres from urban and rural districts of Thua Thien Hue Province, Vietnam. Mother-infant dyads one to six months postpartum were invited to participate. Questionnaires from 431 mothers (urban *n* = 216; rural *n* = 215) assessed demographic and family characteristics, traditional confinement practices, son preference, infant health and social capital. The Edinburgh Postnatal Depression Scale (EPDS) and WHO5 Wellbeing Index indicated depressive symptoms and emotional wellbeing. Data were analysed using general linear models.

**Results:**

Using an EPDS cut-off of 12/13, 18.1 % (*n* = 78, 95 % CI 14.6 - 22.1) of women had depressive symptoms (20.4 % urban; 15.8 % rural). Contrary to predictions, infant gender and traditional confinement were unrelated to depressive symptoms. Poverty, food insecurity, being frightened of family members, and intimate partner violence increased both depressive symptoms and lowered wellbeing. The first model accounted for 30.2 % of the variance in EPDS score and found being frightened of one’s husband, husband’s unemployment, breastfeeding difficulties, infant diarrhoea, and cognitive social capital were associated with higher EPDS scores. The second model had accounted for 22 % of the variance in WHO5 score. Living in Hue city, low education, poor maternal competence and a negative family response to the baby lowered maternal wellbeing.

**Conclusions:**

Traditional confinement practices and son preference were not linked to depressive symptoms among mothers, but were correlates of family relationships and wellbeing. Poverty, food insecurity, violence, infant ill health, and discordant intimate and family relationships were linked with depressive symptoms in Central Vietnam.

## Background

This study sought to add insight into the prevalence of postnatal depressive symptoms and emotional wellbeing in rural Vietnam, and investigates the influence of social factors, including confinement practices and son preference. A burgeoning body of evidence from low-and middle-income (LAMI) countries consistently reveals a similar or higher prevalence of common perinatal mental disorders (CPMDs) compared to high-income-countries (HIC) [1, 2]. In LAMI countries, it is estimated 15.6 % of pregnant women, and 19.8 % of postnatal women experience CPMDs compared with around 10 % and 10-16 % respectively in HIC [1, 3]. Two studies from the North and South of Vietnam report that around one third of women experience a CPMD [4, 5]. The impact of CPMDs is largely unrecognized in international efforts to improve maternal and child health. CPMDs influence a mother’s receptivity and emotional responsiveness to her infant, which is linked to lower birth weights, more diarrhoeal disease, under nutrition and incomplete immunization in LAMI countries [6, 7].

Women in LAMI countries who experience gender-based violence, have little reproductive autonomy, or an unintended or unwanted pregnancy are at higher risk of CPMDs [6]. Bearing a female infant is associated with CPMDs in some LAMI countries, and in China the mothers of daughters are twice as likely to suffer postnatal depression [6, 8]. Son preference is prominent in Vietnam and stems from a convergence of Confucian ideals of family structure and ancestor veneration [9]. The national ‘two child policy’ penalises families with more than two children [10]. Qualitative studies from Vietnam suggest son preference influences the risk of postnatal depression, however no quantitative research has investigated this link [11, 12].

Vietnamese mothers commonly observe a postpartum confinement period where they are cared for by female relatives. Confinement is believed to restore the woman’s strength after birth, and includes practices such as enforced rest, lying over heat, not bathing or washing hair, and avoiding particular foods [4, 13]. It was previously theorised that postpartum confinement was protective against postnatal depression, because it offered women a time of recuperation and social support, and honoured the role of the mother [13, 14]. Evidence from Chinese cultures and one study from Vietnam are inconclusive regarding confinement practices being either risk or protective factors for CPMDs [4, 15]. This study had two objectives: 1.) To estimate the prevalence of postnatal depressive symptoms amongst women in Central Vietnam and 2.) To explore the influence of social, cultural and infant factors on the postnatal emotional wellbeing of women in a culturally distinct province of Vietnam.

## Methods

### Study setting

This study was conducted in Thua Thien Hue Province, Central Vietnam, which has nine districts including the Provincial capital of Hue City. In 2012 the population of Thua Thien Hue was approximately 1.1 million and the per capita income was $1300USD per year [16, 17]. Hue City is a centre for education, health and social services, tourism and light industry. In the rural districts of the province, the main industries are rice farming and aquaculture. In Vietnam, over 90 % of women have a skilled attendant at birth [5]. There is no routine screening for CPMDs in health services.

### Study design

A cross-sectional survey was conducted using a two-stage sampling procedure: 1.) Four districts (two rural and two urban) within Thua Thien Hue Province were randomly selected for inclusion in the study. These were Phong Dien and Phu Vang (rural) and the North and South of Hue City (urban) 2.) Within the included districts, six urban and six rural communes were randomly selected. Each selected commune has one government Commune Health Centre (CHC) that was used as a recruitment point. The survey was conducted between June and October 2010. The survey questionnaire was translated into Vietnamese, back translated to English and subject to consensus panel review by eight bilingual experts according to the method of Brislin [18]. The consensus panel ensured the content of each item was relevant, unambiguous, and framed in a culturally sensitive manner. The questionnaire was then piloted with 30 postpartum women to finalise translation and refine interview procedures.

### Participant selection and recruitment

All postpartum women registered with the selected CHCs who were between one to six months postpartum were eligible to participate. Women less than 18 years old, who delivered babies with congenital abnormalities, or experienced a still birth were excluded in accordance with study ethics approval. Trained data collectors conducted interviews at CHCs, either by appointment or on monthly maternal health days. Some women were interviewed in their homes at their request due to childcare commitments.

### Sample size calculation

A sample size was calculated based on the measurement of clinically significant difference between groups from prior studies using the EPDS in Vietnamese populations from Ho Chi Minh City and Sydney [4, 19]. This required 210 participants per group (rural/urban). Twenty more participants were added to each group in order to accommodate for participant refusal (460 in total).

When calculating sample size, the risk of bias when choosing a sample based on geographical clusters (communes) was considered. Two previous prevalence studies of depression amongst mothers and adults in Vietnam that also used randomly selected communes as a sampling strategy. These studies concluded there were no significant differences in the characteristics of participants between communes [5, 20]. A study of depression amongst 1,976 adults from randomly selected communes in Hue city similarly found no substantial clustering effect (design effect of one, Intra-Class Correlation close to zero 0.0057) [20]. Based on this evidence, demographic homogeneity between communes was assumed when calculating the sample size for this survey.

#### Outcome measures: depressive symptoms and wellbeing

The 10-item Vietnamese version of the Edinburgh Postnatal Depression Scale (EPDS) described by Tran et al. [21] was used to measure depressive symptoms. Existing literature is inconclusive regarding an appropriate cut-off for Vietnamese versions of the EPDS [4, 19, 21]. However, a large systematic review suggests EPDS cut-offs of 12/13 for probable depression are robust for screening in most languages [22].

The five item WHO-5 Wellbeing Index was used to measure positive wellbeing, vitality and general interest in things. WHO5 scores below 13 indicate low wellbeing [23].

### Exposure variables measured

#### Demographics and Socioeconomic Status (SES)

Socio-demographic variables included age, marital status, ethnicity, education level, professional occupation of the mother and her husband, and access to paid maternity leave. Poverty (yes/no) was classified according to the Government Statistics Office (GSO) criterion of a monthly expenditure of 280,000VND (approximately 20USD) or less [17]. Food insecurity was assessed with one question asking how many months during the past year participants could not afford to buy the food their family needed.

#### Maternal and child health

A short checklist of obstetric history included items about mode of delivery, gravidity, parity, infant gender, and birth order. Preference for infant gender (whether the gender of the infant matched the preference the mother had during pregnancy) (preferred, no preference, not preferred), and perceived family reactions to the infant (own mother, husband, mother-in-law) were also assessed (negative/positive). Place of delivery was recorded, as well as previous use of psychiatric services. Episodes of infant diarrhoea and acute respiratory infections (ARIs) in the past two weeks were documented [24]. Weight and length of infants was measured using WHO issued measuring boards and balance scales sensitive to 100g. Questions about exclusive breastfeeding, breastfeeding problems and frequency of child crying were included. A 14-item maternal competence scale measured women’s sense of confidence in their maternal role [25].

#### Confinement period

A checklist of nine traditional confinement and dietary practices was included, as well as a short answer section for women to name other practices they had undergone. Women were asked if there was agreement or conflict between themselves and carers over postpartum confinement practices. Satisfaction with postpartum body image was measured with a likert scale (unsatisfied, no opinion, satisfied).

#### Social support and social capital

The quality of relationships with the participant’s mother, husband, and parents in law was assessed, and a Vietnamese version of the twelve-item Multidimensional Scale of Perceived Social Support (MSPSS) was included [19, 26]. Social Capital was measured using the Short-form Adapted Social Capital Questionnaire (SASCAT) from De Silva et al. [27] which includes social group membership, civic engagement, and five items regarding individual ‘cognitive’ social capital. Women were asked if they had been hit, slapped, kicked, pushed or otherwise physically hurt by their husband in the past twelve months. Two items enquired if women had felt frightened of their intimate partner, or another family member in the past twelve months as proxy measures of emotional abuse.

#### Ethics approval

Ethics approval was obtained from Queensland University of Technology in Australia, and Hue University of Medicine and Pharmacy in Vietnam. Written informed consent was obtained from all participants.

#### Data management and analysis

Statistical analysis was performed using PAWS Statistics version 18.0 and WHO Anthro (version 3.2.2) for anthropometric data [28, 29]. The first aim of this study was to estimate the prevalence of depressive symptoms amongst women in Central Vietnam. Univariate analysis using the EPDS cut-offs suggested by Gibson et al. [22] were used to report prevalence of depressive symptoms. A WHO5 cut-off of 13 was used to report prevalence of wellbeing.

The second aim of this study was to explore what social, cultural and infant influence depressive symptoms and wellbeing amongst women in Central Vietnam. Two multivariable general linear models (GLM) were built, using EPDS score and WHO5 score as continuous outcome variables. Multivariable models were built using Bursac et al’s “Purposeful Selection of Covariates” method [30]. This method has been identified as the most systematic way of determining variable inclusion in linear models where the analyst is interested in identifying risk factors. Hence it is appropriate where the purpose of modeling is exploratory, and uses data from an observational study [31]. This method required the following series of steps: 1.) Bivariate analysis of all independent variables with the dependent variable (EPDS score/WHO5 score) was undertaken. All covariates at the alpha level of <0.25 or less were included in an initial multivariable model. The socio-demographic characteristics of age (years), education (years), location (urban/rural), socioeconomic status (classed by the government as poor), and parity (1, 2, ≥ 3 children) were also included for the purposes of adjustment. The rationale for this modest level of significance is based on recommendations for linear regression by Bendel and Afifi [32]. 2.) All covariates that were not significant at *p* <0.25 and not a confounder were then removed from the model. Confounders were classified as any variable that changes the coefficient of another variable in the model by 15-20 % [33]. A change in a parameter estimate above this level indicates that the excluded variable provides a needed adjustment for one or more of the other variables in the model. 3.) A reduced model was then fit with all remaining variables, and variables that were either not significant at the 0.1 alpha level or met the criteria for confounders were again removed. 4.) One at a time, all covariates excluded from the initial model fits (steps 2 and 3) are re-tested to confirm they are neither statistically significant (*p* ≤ 0.05) or a confounder. At the end of this process, significant covariates and confounders are added back into the model, and assumptions and goodness of fit criteria are checked [33]. In Model One, the retained confounders were days of confinement, body image, and carer attitudes to confinement. In Model Two no confounders were retained.

## Results

A total of 777 mothers from selected communes were identified as eligible and contacted about the study, of which 465 responded and were recruited and included in the study. This indicated a refusal rate of 40.15 %, and a sufficient sample size (>210 per group) was met. After data collection 34 questionnaires were excluded before analysis (due to questionnaires being >10 % incomplete), resulting in a total of 431 participants. The final sample included 216 urban women and 215 rural women.

The EPDS cut-offs for PND suggested by Gibson et al., [22] yielded prevalence estimates of 37.1 % for possible depression (*n* = 431, 95 % CI 32.5–41.9) and 18.1 % (*n* = 431, 95 % CI 14.6 – 22.1) for probable depression. Sixty-three women (14.7 %) (*n* = 429, 95 % CI 11.5–18.4) had WHO5 scores indicating low wellbeing. There was satisfactory convergent validity between the EPDS and WHO5 scores (R_s_ -0.514). The socio-demographic and reproductive health characteristics of participants are outlined in Table 1. Women had a mean age of 29.45 years (SD 6.03). There was a significant negative correlation between years of education and EPDS score. The mean number of pregnancies per woman was 2.08 (SD 1.34), with multiple pregnancies being more common in rural areas. Six women, (1.5 %) reported they had previously had an infant die at birth or in the first week of life. In terms of economic disadvantage, 9.2 % (*n* = 39) of women were in a family classified by the government as poor. A similar proportion of women in both urban and rural areas were classified as poor, with 8.4 % (*n* = 18) and 9.9 % (*n* = 21) respectively.

**Table 1:** Sociodemographic and reproductive health characteristics

Variable	N (%)	Mean EPDS score	SD	Test statistic^a^	DF (One-Way ANOVA)	Mean WHO5 Score	SD	Test Statistic^a^	DF (One-Way ANOVA)
Location									
Urban	216 (50.1)	7.72	5.28	-0.689		16.78	5.09	-0.221	
Rural	215 (49.9)	8.07	5.04			16.84	4.59		
Marital Status									
Married	419 (97.2)	7.72	5.01	-4.12**		16.85	4.87	2.64*	
Not Married	12 (2.8)	13.83	6.75			14.64	2.77		
Husband Employed									
Yes	408 (94.7)	7.66	4.93	9.686**	2	16.88	4.86	1.48	2
No	10 (2.3)	9.50	5.84			15.70	5.43		
Not Applicable	13 (3.0)	13.77	7.15			14.67	3.14		
Family Classed as Poor									
Yes	39 (9.2)	11.00	5.36	4.004**		14.28	5.92	-3.43*	
No	387 (90.8)	7.58	5.06			17.04	4.65		
Paid Maternity Leave									
Yes	91 (21.1)	6.97	4.43	-1.93*		18.39	5.29	3.58**	
No	340 (78.9)	8.14	5.31			16.37	4.63		
Food Insecurity (months per year)									
Never	237 (55.2)	6.49	5.48	8.62**	5	17.55	4.69	3.23*	5
Every month	39 (9.0)	10.46	5.04			16.38	4.88		
Every 1-3 months	50 (11.7)	9.76	5.87			15.20	5.38		
Every 3-6 months	49 (11.4)	9.38	5.15			15.45	4.85		
Every 6-9 months	34 (7.9)	8.79	4.86			16.41	4.44		
Once a year	20 (4.6)	8.9	6.12			16.10	4.39		
Mother’s Occupation									
None	72 (17.1)	8.55	5.24	2.629*	4	16.14	4.55	7.97**	4
Unstable manual	163 (37.9)	8.56	5.45			15.91	4.95		
Stable manual	109 (25.9)	7.5	4.95			16.55	3.91		
Professional, no qualifications	9 (2.1)	8.00	6.34			16.78	4.09		
Professional with qualifications	67 (15.9)	6.37	4.21			19.58	4.90		
Husband’s Occupation									
None	15 (3.5)	11.8	7.63	5.975**	4	13.93	4.58	7.35**	4
Unstable manual	192 (45.3)	8.58	5.28			15.79	4.75		
Stable manual	132 (31.1)	7.02	4.41			17.39	4.26		
Professional, no qualifications	14 (3.3)	8.57	5.17			15.71	5.97		
Professional with qualifications	71 (16.7)	7.83	4.51			19.21	4.74		
Parity									
1	208 (48.2)	7.41	5.07	1.746	2	17.29	4.74	2.78	2
2	120 (27.8)	8.32	4.81			16.64	4.52		
≥ 3	103 (23.9)	8.35	5.67			15.94	5.30		
Birth mode									
Vaginal	287 (70)	7.61	4.97	-0.26		16.65	4.44	-1.79	
Caesarean section	123 (30)	7.78	5.16			17.57	5.35		
Infant diarrhoea									
Yes	45 (10.5)	10.76	5.73	3.96**		15.62	4.39	-1.67	
No	383 (89.4)	7.58	4.99			16.89	4.87		
Infant ARI									
Yes	46 (10.7)	9.04	6.86	1.55		15.91	5.07	-1.24	
No	381 (89.2)	7.79	4.92			16.85	4.79		
Breastfeeding problems									
Yes	123 (29.6)	9.77	5.50	4.58**		15.63	5.14	-2.83*	
No	292 (71.3)	7.29	4.83			17.09	4.61		

^**a**^T-test or One-Way ANOVA

*p = 0.05-0.001

**p <0.001

### Confinement practices

Notably, all women followed at least one traditional confinement practice. The mean number of days spent in confinement was 57.56 (SD 34.518, range 0–180). The prevalence of different confinement practices and associations with EPDS scores are shown in Table 2. There was a correlation between number of days in confinement and EPDS score (R_s_ 0.19, *p* <0.001). The woman’s own attitude towards the importance of confinement was not significant (t 1.75, mean difference −0.89 95 % CI−1.88 – 0.08 p = 0.08). However, the attitude of their main caregiver regarding the importance of confinement (not important, important) was significantly related with EPDS score (t – 2.25, mean difference −1.15 95 % CI −2.15 – 10.15 p = 0.03).

**Table 2:** Traditional confinement practices reported by women in urban and rural areas

Confinement Practice	Urban		Rural		Total		Mean EPDS Score	SD	Test Statistic	*P* value
	*N*	*%*	*N*	*%*	*N*	*%*	Practiced			
							Not practiced			
Lying over heat	150	69.4	183	85.1	333	77.3	8.1	5.07	1.53	N.S
							7.2	5.41		
Cotton in ears	191	88.4	195	90.7	386	89.6	7.9	5.24	0.95	N.S
							7.2	4.38		
Not watching TV	73	33.8	117	54.4	190	44.1	8.5	5.35	2.31	0.02
							7.4	4.97		
No bathing (1-3 months)	120	55.6	146	67.9	266	61.7	8.2	5.35	2.03	0.04
							7.3	4.78		
Using traditional medicine	134	62	136	63.3	270	62.6	7.3	5.29	-2.92	0.04
							8.8	4.80		
Stomach binding	23	10.6	22	10.2	45	10.4	8.4	6.10	0.76	N.S
							7.8	5.04		
Not washing Hair	103	47.7	137	63.7	240	55.7	8.4	5.46	2.35	0.02
							7.2	4.69		
Not reading	74	34.3	111	51.6	185	42.9	8.4	5.45	2.04	0.04
							7.5	4.89		
Eating special foods	146	67.6	129	60.6	275	64.1	7.3	5.17	-3.45	<0.001
							9.1	4.94		
Avoiding proscribed foods	168	78.5	173	80.5	341	79.5	7.8	5.26	-0.75	N.S
							8.3	4.77		

### Son preference

One-way ANOVAs explored parity, maternal preference of infant gender during pregnancy, and gender of the last infant, and none were significantly associated with depressive symptoms. The number of male or female children a woman had was not significantly related to EPDS score (F (3, 430) = 1.40; 5, p 0.24 and F (3, 430) = 1.87, p = 0.149 respectively). There was no significant difference in EPDS score between different child gender combinations (all boys, all girls, boys and girls) in multiparous women (*n* = 223) (F (2, 223) = 2.302, p = 0.78). The number of female children a woman had was significantly associated with lower WHO5 wellbeing scores (F, (3, 430) = 2.92, p = 0.03).

### Intimate partner, family and social relationships

In bivariate analysis, the four items regarding cognitive social capital (which indicates feeling part of the community and being able to trust community members) were all associated with EPDS score with a cut-off of 12/13 (*p* <0.05). However, indicators of structural social capital (participation in groups and civil action) were not significantly associated with depressive symptoms. The majority (88.3 %, *n* = 380) of participants said their relationship with their mother was very close, 89.6 % (*n* = 381) said their relationship with their husband was very close and 56.4 % (*n* = 239) described a very close relationship with their mother-in-law. Depressive symptoms and scores on the MSPSS were negatively correlated (R_s_ -0.237, *p* <0.001), as were poor relationships with husbands (t −3.74, mean difference −9.33 95 % CI −14.23 - -4.43 *p* < 0.001), and mothers-in-law (t −2.31, mean difference −2.43 95 % CI −4.5 - -0.36 p = 0.02). At the bivariate level, it appeared that as women’s levels of social support increased, their EPDS scores decreased. Negative reaction of relatives to the infant, are reported in Table 3. If a woman had negative reactions to the infant from her husband (*p* = 0.05), the woman’s own mother (p = 0.02) and her mother in law (*p* = 0.01) were all associated with higher EPDS score in bivariate analysis, as were intimate partner violence, and being frightened of your husband or family (*p* < 0.001). Other findings regarding family relationships and support are shown in Table 1 and Table 3*.*

**Table 3:** Infant gender, reactions of family members to infant and family relationships

Variable	N (%)	Mean EPDS score (with 95 % CI for ANOVA)	Mean Difference (95 % CI) For t-tests	Test statistic^a^
Gender of last infant				
Male	233 (54)	7.64	−0.54 (−1.52 – 0.44)	1.089
Female	198 (46)	8.19		
Maternal Preference for Infant Gender^c^				
Gender preferred	256 (59.7)	7.63 (6.9 – 8.2)	5.29	2.144^b^
Gender not preferred	71 (16.6)	9.04 (7.7 – 10.3)	5.59	
No preference	101 (23.6)	7.73 (6.9 – 8.3)	4.46	
Frightened of Husband				
Yes	41 (9.7)	11.15 (9.4 – 12.8)		24.65**^b^
Sometimes	109 (25.5)	9.36 (8.5 – 10.1)		
No	271 (64.3)	6.58 (6.0 – 7.1)		
Frightened of Family				
Yes	45 (10.4)	10.73 (8.9 – 12.5)		19.07**^b^
Sometimes	82 (19.1)	9.82 (8.9 – 10.7)		
No	302 (70.3)	6.95 (6.4 – 7.5)		
Intimate Partner Violence				
Yes	27 (5.4)	13.33	5.91 (4.02 – 7.80)	6.15**
No	396 (93.6)	7.41		
Own Mother’s Reaction to the Infant				
Negative	23	10.26	2.56 (0.04 – 4.7)	2.32*
Positive	394	7.70		
Mother in Law’s Reaction to the Infant				
Negative	31	10.03	2.46 (0.62 – 4.29)	2.62**
Positive	382	7.57		
Husband’s Reaction to the Infant				
Negative	23	9.78	2.12 (0.018 – 4.24)	1.98*
Positive	400	7.65		

^**a**^T-test or One-Way ANOVA

*p = 0.05-0.001

**p <0.001

^b^One-Way ANOVA, degrees of freedom = 2

^c^This explores whether the infant gender the mother preferred in pregnancy was the same as infant gender at birth

### Infant health

The associations between EPDS scores and infant health are shown in Table 1. The amount a child cried, breastfeeding difficulties, and diarrhoea in the past two weeks were all significantly associated with depressive symptoms. In terms of the anthropometric data, EPDS score was associated with stunting (low length for age) (mean EPDS score 9.3 stunting, 7.6 no stunting) (t – 2.23, p = 0.03). Other measures of malnutrition (weight for length and length for age) were not significant. Women who had less maternal competence (based on the Maternal Competence Scale (MCS)] were more likely to have a high EPDS score (Pearson’s R −0.105, p = 0.03).

### Health care utilization

Many women gave birth in a tertiary hospital (45.5 %, *n* = 196). This was more common for urban (60.2 %, *n* = 130) compared to rural women (30.7 %, *n* = 66). Rural women were more likely than urban women to give birth in a district hospital (34 %, *n* = 73 vs. 13.4 %, *n* = 29) or a CHC (24.2 %, *n* = 52 vs. 6.5 %, *n* = 14). Only 1.2 % of women (*n* = 5) reported that they had ever been treated for a psychiatric illness.

### Multivariable models

#### Model One: EPDS score

The first model as a whole accounted for 30.2 % of the variance in EPDS score. After adjustment for socio-cultural variables (age, location, education, and parity) the statistically significant variables remaining in the model are described in Table 4. These variables were: being classed as poor, food insecurity, experiencing violence in the past 12 months, being frightened of family members, being frightened of your husband, husband’s work status, breastfeeding difficulties, diarrhoea, and cognitive social capital. The number of days in confinement, body image and carer attitudes to confinement practices remained in the model as confounders. Infant gender was not retained in the final fitting of this model.

**Table 4:** Multivariable model of EPDS Score

Parameter	Marginal Mean (95 % CI)	N	B	Sig.
Intercept			6.688	.001
Socio-Demographic Characteristics				
Location				
Urban	13.80 (12.06 - 15.56)	193	.357	.449
Rural	13.45 (11.66 - 15.24)	194	0^a^	
Classed as Poor				
Yes	14.47 (12.30 – 16.63)	33	1.679	.039
No	12.79 (11.24 – 14.34)	354	0^a^	
Husband employed				
No	14.84 (12.25 – 17.44)	15	2.429	.036
Yes	12.41 (11.12 – 13.71)	372	0^a^	
Food Insecurity				
Every month	14.81 (12.66 – 16.97)	35	2.270	.007
Every 1–6 months	14.19 (12.39 – 16.00)	93	1.655	.005
Every 6–12 months	12.96 (10.94 – 14.99)	46	.425	.567
Never	12.54 (10.70 – 14.38)	213	0^a^	
Education (years)			.019	.768
Age (years)			.070	.147
Parity				
1	13.56 (11.75 – 15.37)	179	.036	.964
2	13.79 (11.99 – 15.06)	113	.272	.694
≥3	13.53 (11.50 – 15.55)	95	0^a^	
Confinement Practices				
Carer’s opinion of confinement				
Important	13.19 (11.36 – 15.02)	144	-.878	.065
Not important	14.07 (12.36 – 15.77)	243	0^a^	
Days in Confinement			-.011	.128
Social Support				
Frightened of Family				
Yes	14.09 (11.94 – 16.23)	38	1.522	.059
Sometimes	14.24 (12.34 – 16.35)	79	1.677	.009
No	12.53 (10.84 – 14.29)	270	0^a^	
Violence in past 12 months				
Yes	15.17 (12.96 – 17.38)	24	3.090	.001
No	12.08 (10.44 – 13.73)	363	0^a^	
Frightened of Husband				
Yes	14.84 (12.74 – 16.93)	37	2.418	.003
Sometimes	13.63 (11.79 – 15.47)	105	1.217	.035
No	13.06 (10.62 – 14.22)	245	0^a^	
Body image				
Unsatisfied	13.40 (11.36 – 15.24)	130	.348	.492
No opinion	14.43 (11.79 – 15.47)	86	1.372	.021
Satisfied	13.06 (12.55 – 16.31)	171	0^a^	
Cognitive Social Capital			−1.058	.000
Infant Health				
Infant diarrhoea in past 2 weeks				
Yes	14.43 (12.40 – 16.47)	41	1.615	.032
No	12.82 (11.16 – 14.48)	346	0^a^	
Breastfeeding Problems				
Yes	14.26 (12.50 – 16.02)	112	1.267	.012
No	12.99 (11.20 – 14.80)	275	0^a^	

^a^This parameter is set to zero because it is redundant

Marginal means indicate differences in EPDS scores within covariates. Notably, there were substantial differences in EPDS scores between never being food insecure, and being food insecure every month; between experiencing violence or not in the past 12 months; and between mothers whose infant had diarrhoea in the past two weeks or not. Women with breastfeeding problems had more depressive symptoms than women who did not. There was a negative relationship between cognitive social capital and EPDS score; each point of cognitive social capital score decreased EPDS score by 1.06 points. The overall model was significant (*p* <0.001) and had a partial eta^2^ of 0.341, indicating the effect size of the overall model was quite large.

#### Model two: WHO5 Wellbeing score

The second model as a whole accounted for 22.1 % of the variance in WHO5 score. There was high inter-item correlation for the answers regarding different relative’s reactions to infant gender (r = 0.88 between husband and own mother’s reactions). Only “reactions by mother-in-law” was left in the model as it resulted in the best fit.

After adjustment for socio-demographic variables (infant gender, location, education, poverty, and parity) the following statistically significant variables were identified: experiencing violence in the past 12 months, being frightened of family members, maternal competence, family (mother-in-law) reacting badly to the baby, and food insecurity (See Table 4). Of the socio-demographic variables adjusted for, being classed by the government as poor remained statistically significant, as did urban location and years of education. Infant gender and parity were not significant.

There were strong negative associations between wellbeing and intimate partner violence in the past 12 months, being frightened of family members in the past 12 months and extended family (mother in law) reacting badly to the infant. Urban and/or impoverished women had lower levels of wellbeing than rural and more affluent women. Education and maternal competence scores were also significant. Participants who were food insecure every 1–6 months had lower wellbeing scores than women who were never food insecure (Table 5).

**Table 5:** Multivariable Model of WHO5 Wellbeing Index

Parameter	Marginal Mean (95 % CI)	N	B	Sig.
Intercept			11.633	.000
Socio-Demographic Characteristics				
Location				
Urban	12.34 (10.96-13.72)	200	−1.111	.015
Rural	13.45 (12.11-14.79)	200	0^a^	
Classed as Poor				
Yes	11.88 (10.09 – 13.66)	36	−2.032	.009
No	13.91 (12.76-15.05)	364	0^a^	
Infant Gender				
Male	12.89 (11.53-14.25)	218	-.011	.980
Female	12.90 (11.55-14.25)	182	0^a^	
Violence in past 12 months				
Yes	11.16 (9.23-13.07)	25	−3.481	.000
No	14.64 (13.54-15.73)	375	0^a^	
Frightened of Family				
Yes	12.15 (10.44-13.86)	39	−1.980	.008
Sometimes	12.41 (10.9 – 12.91)	80	−1.717	.002
No	14.13 (12.79 – 15.47)	281	0^a^	
MIL reaction to infant				
Negative	11.64 (9.83-13.48)	29	−2.483	.004
Positive	14.14 (12.96-15.31)	371	0^a^	
Parity				
1	13.13 (11.75-14.5)	193	.544	.371
2	12.98 (11.56-14.40)	112	.400	.524
> = 3	12.58 (11.04-14.12)	95	0^a^	
Education (years)		400	.188	.002
MCS Score		400	.135	.000
Food Insecurity				
Every month	13.82 (12.03-15.61)	34	1.018	.222
Every 1–6 months	11.70 (10.21-13.19)	92	−1.099	.055
Every 6–12 months	13.27 (11.64-14.89)	49	0.469	.511
Never	12.70 (11.33-14.26)	225	0^a^	

^a^This parameter is set to zero because it is redundant

## Discussion

To our knowledge, this is the first study of postnatal mental health in urban and rural central Vietnam. The estimated prevalence of 18.1 % for probable depressive symptoms is lower than observed in Ho Chi Minh City (33 %) and Northern Vietnam (29.9 %) [4, 5]. This difference could be due to the use of a conservative EPDS cut-off or differences in participant disclosure. However, the prevalence in Thua Thien Hue is similar to the summative estimate from a systematic review of perinatal mental disorders in LAMI countries (19.8 %, 95 % CI 19.5-20) [1]. This provides further evidence that postnatal depressive symptoms are prevalent throughout Vietnam, including the central provinces. This study also aimed to explore the social, cultural and infant factors associated with depressive symptoms amongst women in Central Vietnam. Depressive symptoms were associated with poverty, food insecurity, violence, infant ill health, and discordant intimate and family relationships. Interestingly, urban women were slightly more likely to have a high EPDS score than rural women. However, in multivariate analysis, poverty and food insecurity were more strongly associated with depressive symptoms and low wellbeing than geographic location. Two cultural factors previously suggested to be influential in the Vietnamese context (traditional confinement practices and son preference) were not linked to depressive symptoms, but poor family relationships and negative reactions to the infant lowered maternal wellbeing.

The reported prevalence of intimate partner violence (IPV) in the past year (6.4 %) was higher than previously observed in Vietnam [5]. In Northern Vietnam, 2 % and 3.1 % of new mothers experienced IPV in rural and urban areas respectively, compared to 5.7 % and 7.1 % in the past year in rural and regional Thua Thien Hue [5]. Thirty five percent of women reported being frightened of their husband, and this was significantly correlated with EPDS scores. Experiencing gender-based violence and having an antagonistic partner increases the risk of CPMDs in other LAMI settings [1]. Around one third (29.6 %) of women had always or sometimes been frightened of their family in the past year, which was much higher than the 6 % found in Northern Vietnam [5, 34].

Despite giving birth to girls being a risk factor in some LAMI countries [1], this study did not find a strong association between infant gender and EPDS score. This included where mothers had multiple children and no sons, and where the infant gender wasn’t preferred. Modest associations between low wellbeing and number of girl children, family reactions to the infant, and perceived maternal competence were observed. The lack of an association between infant gender and depressive symptoms was unexpected considering that societal preference for sons is well documented in Vietnam [11, 12]. Not having a son in the first two live births may cause economic pressure not only through the increased expense of a third child but also by affecting family status and occupational prestige [10]. The findings of this study may have been limited by the fact that around half of the participants were primiparous, which meant they had at least one more opportunity to have a son. The smaller sample size of multiparous women prohibited further subgroup analysis. However, despite no associations being found between PND, infant gender or parity, the mother’s perception of the reaction of relatives to the infant were significantly associated with both EPDS and WHO5 scores. Associations between the reactions of other relatives to the infant and maternal depression have also been noted in China. Particularly, Xie et al., [35] suggested that the link between PND and infant gender found in their study was not due to the infant gender per se, but rather the social context and reactions of relatives to the baby. Also in India it was noted some relatives would treat a girl baby differently, whether overtly or subtly [36, 37].

Intense levels of postpartum support from relatives have been described as a ‘double edged sword’ [38]. Whilst the majority of women in Hue said they had a good relationship with their mother (88.3 %) and mother-in-law (56.4 %), women with more distant interpersonal feelings may still have had this relative as their postnatal caregiver. Being in close proximity to a disapproving or emotionally cold relative during confinement would negatively affect emotional wellbeing. Having belligerent in-laws and poor family relationships are identified risk factors for CPMD in other LAMI countries [1]. In this study, being frightened of family members was significantly associated with more depressive symptoms and poorer wellbeing in multivariable analysis. This points to women living in a situation of exposure to emotional abuse and social adversity. Also, if a relative (in particular, mother-in-law) had negative reactions to the infant, this significantly decreased wellbeing.

Thua Thien Hue is regarded as one of the more traditional provinces in Vietnam, and was the ancient capital [39]. This may in part explain the greater popularity of certain confinement practices compared to a similar study in Ho Chi Minh City [4]. Studies regarding confinement and CPMDs have yielded mixed results and to date, no particular confinement practice is consistently associated with depressive symptoms [15]. In central Vietnam, no confinement practice remained significant in multivariable analysis, nor did number of days in confinement. It appears that rather than having an independent protective effect, it is the quality of support provided by female relatives during confinement that influences emotional wellbeing.

Depressive symptoms were more common among women reporting breastfeeding problems and infant diarrhoea. The symptoms of CPMDs, such as fatigue, reduce a woman’s ability to emotionally respond to infants and children, maintain a clean environment, and compete for limited health resources when their child is ill [2]. In LMIC, the negative effect of CPMDs on the growth and development of infants and children is independent of poverty, malnutrition and chronic social hardship [6, 7].

Limitations of this study include those inherent with using a cross-sectional design. However a randomly selected, community based sample resulted in a more representative sample of postpartum women in central Vietnam than a facility based survey. The temporal limitations of cross-sectional studies prohibited the direction of associations from being determined in a due to the chronic remitting and relapsing nature of depression. Potential correlates such as antenatal or previous episodes of depression could not be measured, as no antenatal mental health data was available through the CHCs. Response bias can also affect the results of studies relying on self-report data. Whilst the response rate was adequate, information on the characteristics of eligible women who did not participate was not available. Studies from HIC show that non-responders are often from lower socioeconomic or educational groups, and may have poorer physical or mental health [40]. This could contribute to an underestimation of prevalence, and introduce bias in associations between social characteristics and depressive symptoms.

## Conclusions

Poverty, food insecurity, low social capital, living in a context of fear, and experience of intimate partner violence were most strongly associated with postnatal depressive symptoms in central Vietnam. Since the commencement of the 2000–2015 Millennium Development Goals (MDGs) an emerging body of research has revealed the high prevalence of maternal mental disorders in LAMI countries, and their detrimental effect on the growth and cognitive development of children [6, 7]. There are also complex multi-directional relationships between CPMDs and poverty, gender inequality, violence and child health [1, 41]. Further research using designs that can determine the direction and strength of these relationships is recommended. More research into effective low-cost interventions for treating CMPDs as well as studies about how to nurture the growth and development of children with family members affected by CMPDs in LAMI countries is required.

## Abbreviations

EPDS: Edinburgh Postnatal Depression Score
WHO-5: WHO-5 Wellbeing Index
LAMI: Low and Middle Income
CPMD: Common Perinatal Mental Disorder
HIC: High Income Country
CHC: Commune Health Centre
SES: Socio-Economic Status
ARI: Acute Respiratory Infection
WHO: World Health Organization
MSPSS: Multimensional Scale of Perceived Social Support
SASCAT: Short-Form Adapted Social Capital Questionnaire
GLM: General Linear Model
SD: Standard Deviation
MCS: Maternal Competence Scale

## References

[1] Fisher J, Cabral de Mello M, Patel V, Rahman A, Tran T, Holton S (2012). Prevalence and determinants of common perinatal mental disorders in women in low-and lower-middle-income countries: a systematic review. Bull World Health Organ.

[2] Fisher J, Cabral de Mello M, Izutsu T, Chandra PS, Herrman H, Fisher J, Kastrup M, Niaz U, Rondon MB, Okasha A (2009). Mental health aspects of pregnancy, childbirth and the postpartum period. Contemporary Topics in Women’s Mental Health: Global perspectives in a changing society.

[3] Beyondblue, The National Depression Initiative (2008). Perinatal mental health national action plan: 2008–2010 full report.

[4] Fisher J, Morrow M, Nhu Ngoc N, Hoang Anh L (2004). Prevalence, nature, severity and correlates of postpartum depressive symptoms in Vietnam. Br J Obstet Gynaecol.

[5] Fisher J, Tran T, Buoi LT, Rosenthal D, Kriitmaa K, Tuan T (2010). Common perinatal mental disorders in women in the north of Vietnam: Community prevalence and interaction with health care use. Bull World Health Organ.

[6] Rahman A, Fisher J, Bower P, Luchters S, Tran T, Yasamy M (2013). Interventions for common perinatal mental disorders in women in low-and middle-income countries: a systematic review and meta analysis. Bull World Health Organ.

[7] Stewart RC (2007). Maternal depression and infant growth: a review of recent evidence. Matern Child Nutr.

[8] Xie R, He G, Bradwejn B, Walker M, Wen SW (2007). Fetal gender and postpartum depression in a cohort of Chinese women. Soc Sci Med.

[9] Santillan D, Schuler S, Anh HT, Minh TH, Mai BTT (2002). Limited equality: Contradictory ideas about gender and the implications for reproductive health in rural Vietnam. J Health Manage.

[10] Pham BN, Hall W, Hill PS, Rao C (2008). Analysis of socio-political and health practices influencing sex ratio at birth in Vietnam. Reprod Health Matter.

[11] Niemi ME, Falkenberg T, Nguyen MTT, Nguyen MTN, Patel V, Faxelid E (2010). The social contexts of depression during motherhood: A study of explanatory models in Vietnam. J Affect Dis.

[12] UNFPA (2012). Recent Change in the Sex Ratio at Birth in Vietnam.

[13] Rice PL (1999). Asia mothers, western birth.

[14] Pilsbury BL (1978). “Doing the month” confinement and convalescence of Chinese women after childbirth. Soc Sci Med.

[15] Wong J, Fisher J (2009). The role of traditional confinement practices in determining postpartum depression in women in Chinese cultures: A systematic review of the English language evidence. J Affect Dis.

[16] Thua Thien Hue Province: Thua Thien Hue Portal [http://www1.thuathienhue.gov.vn/]

[17] Government Statistics Office of Vietnam: Statistical Censuses & Surveys [www.gso.gov.vn/default_en.aspx?tabit=491]

[18] Brislin RW (1990). Applied Cross-cultural Psychology.

[19] Stuchbery M, Matthey S, Barnett B (1998). Postnatal depression and social supports in Vietnamese, Arabic and Anglo-Celtic mothers. Soc Psychiatry Psychiatr Epidemiol.

[20] Doan VDK: What explains the association between socioeconomic status and depression among Vietnamese adults? PhD Thesis, Queensland University of Technology, School of Public Health; 2011. http://eprints.qut.edu.au/view/person/Doan,_Vuong.html

[21] Tran T, Tran T, La BT, Lee A, Rosenthal D, Fisher J (2011). Screening for perinatal common mental disorders in women in the north of Vietnam: A comparison of three psychometric instruments. J Affect Dis.

[22] Gibson J, McKenzie-McHarg K, Shakespeare J, Price J, Gray R (2009). A systematic review of studies validating the Edinburgh Postnatal Depression Scale in antepartum and postpartum women. Acta Psychiatr Scand.

[23] World Health Organisation: WHO-Five Well-being Index (WHO-5) [http://www.who-5.org]

[24] UNICEF: Child Survival. [www.unicef.org/sapc08/]

[25] Liu C, Chen YC, Yeh Y, Hsieh YS (2012). Effects of maternal confidence and competence on maternal parenting stress in newborn care. J Adv Nurs.

[26] Glazier R, Elgar F, Goel V, Holzapfel S (2004). Stress, social support and emotional distress in a community sample of pregnant women. Journal of Psychosom Obstet Gynecol.

[27] De Silva MJ, McKenzie K, Harpham T, Huttly SR (2005). Social capital and mental illness: a systematic review. J Epidemiol Community Health.

[28] PASW (Predictive Analytic Software): Statistical Package for the Social Sciences (version 18.0). [Computer Software]; 2009.

[29] World Health Organisation: WHO Anthro (version 3.2.2, January 2011) [Computer Software]; 2011.

[30] Bursac Z, Gauss CH, Williams DK, Hosmer DW (2008). Purposeful selection of variables in logistic regression. Source Code Biol Med.

[31] Gregg MB (2008). Field Epidemiology.

[32] Bendel RB, Afifi AA (1977). Comparison of stopping rules in forward regression. J Am Stat Assoc.

[33] Hosmer DW, Lemeshow S, May S (2008). Applied survival analysis: Regression modeling of time-to-event data (2nd ed).

[34] Tran TD, Biggs BA, Tuan T, Casey G, Hanieh S, Simpson J (2014). Psychological and social factors associated with late pregnancy inron deficiency anaemia in rural Viet Nam: A population-based prospective study. PLoS One.

[35] Xie R, He G, Koszycki D. Fetal sex, social support and postpartum depression. Canadian J Psychiatr, 2009;54(12):856–856. 10.1177/070674370905401105 19961663

[36] Jha P, Kesler MA, Kumar R, Ram F, Ram U, Lukasz A (2011). Trends in selective abortions of girls in India: analysis of nationally representative birth histories from 1990 to 2005 and census data from 1991 to 2011. Lancet.

[37] Patel V, Rodrigues M, DeSouza N (2002). Gender, poverty, and postnatal depression: a study of mothers in Goa. India. Am J Psychiatr.

[38] Lee DTS, Yip ASK, Leung TYS, Chung TKH (2004). Ethnoepidemiology of postnatal depression: Prospective multivariate study of sociocultural risk factors in a Chinese population in Hong Kong. Br J Psychiatry.

[39] Doan VH, Nguyen VT (2002). Traditional and Modern Medicine. An introduction to Vietnam and Hue.

[40] Linden-Bostrom M, Persson C (2012). A selective follow-up study on a public health survey. Eur J Pub Health.

[41] Patel V, Araya R, Lima M, Ludermir A, Todd C (1999). Women, poverty and common mental disorders in four restructuring societies. Soc Sci Med.

